# Estimation of Chlorophyll Fluorescence at Different Scales: A Review

**DOI:** 10.3390/s19133000

**Published:** 2019-07-08

**Authors:** Zhuoya Ni, Qifeng Lu, Hongyuan Huo, Huili Zhang

**Affiliations:** 1 Key Laboratory of Radiometric Calibration and Validation for Environment Satellites, National Satellite Meteorological Center, China Meteorological Administration, Beijing 100081, China; 2 College of Architecture and Civil Engineering, Beijing University of Technology, Beijing 100124, China; 3 Jiangxi Technical College Of Manufacturing, Nanchang 330095, China

**Keywords:** chlorophyll fluorescence, Fraunhofer lines, physically-based method, statistically-based method

## Abstract

Measuring chlorophyll fluorescence is a direct and non-destructive way to monitor vegetation. In this paper, the fluorescence retrieval methods from multiple scales, ranging from near the ground to the use of space-borne sensors, are analyzed and summarized in detail. At the leaf-scale, the chlorophyll fluorescence is measured using active and passive technology. Active remote sensing technology uses a fluorimeter to measure the chlorophyll fluorescence, and passive remote sensing technology mainly depends on the sun-induced chlorophyll fluorescence filling in the Fraunhofer lines or oxygen absorptions bands. Based on these retrieval principles, many retrieval methods have been developed, including the radiance-based methods and the reflectance-based methods near the ground, as well as physically and statistically-based methods that make use of satellite data. The advantages and disadvantages of different approaches for sun-induced chlorophyll fluorescence retrieval are compared and the key issues of the current sun-induced chlorophyll fluorescence retrieval algorithms are discussed. Finally, conclusions and key problems are proposed for the future research.

## 1. Introduction

Since the 1980s, vegetation chlorophyll fluorescence has been an effective, non-destructive, and direct way to monitor changes in the physiological state of vegetation [1,2]. Chlorophyll fluorescence is excited by photosynthetic tissue under the sun’s illumination, producing a spectrum ranging from 640–800 nm, with two peaks centered at 685 nm and 740 nm [3,4,5]. Due to the direct and close relationship between photosynthesis and chlorophyll fluorescence [6,7,8,9,10,11], remote sensing of chlorophyll fluorescence can be used to derive gross primary productivity (GPP) [9,12,13,14,15,16,17,18,19,20,21,22,23]. Therefore, the chlorophyll fluorescence emission can be used as an indicator of photosynthesis.

Solar-induced fluorescence (SIF) retrieval methods are developed based on the fluorescence signal filling in the Fraunhofer lines or oxygen absorption bands [24]. Near the ground, SIF retrieval includes the active [25,26,27,28,29,30,31,32] and passive measurement. To extend the near-surface SIF inversion algorithm to the satellite platform, accurate atmospheric correction information is required in order to obtain fluorescence radiance values. Since accurate atmospheric parameters are difficult to obtain, the near-surface SIF retrieval algorithm has poor applicability for satellite platforms. Since the first global SIF map was produced [33,34,35], many researchers have developed SIF inversion methods from satellite data and have successfully extracted SIF from GOSAT, GOME-2, OCO-2, SCIAMACHY, and TanSat data [36,37,38,39,40,41,42,43]. These methods are mainly divided into two approaches: physical approach and statistical approach. These two techniques mainly use Fraunhofer dark line or oxygen absorption bands as the retrieval window, including the physical methods [33,36,38,44,45,46,47,48] and statistical methods [34,37,38,43,49,50,51]. The SIF retrieval methods have been successfully applied in the above-mentioned satellite data. To deeply understand the function of chlorophyll fluorescence in vegetation, the fluorescence explorer project (FLEX) was developed. The FLEX mission will map vegetation fluorescence to quantify photosynthesis activity. The FLORIS sensor will be in orbit in tandem with one of the Copernicus Sentinel-3 satellites, which will be launched in 2022 [52].

In recent years, the reviews of sun-induced fluorescence have been published. Maxwell et al. introduced the method and application of chlorophyll fluorescence in field and laboratory situations [53]. Meroni et al. summarized the sun-induced chlorophyll fluorescence (SIF) retrieval methods including the radiance-based methods and reflectance-based methods and its application at different scales [5]. Cendrero-Mateo et al. summed up the active and passive chlorophyll fluorescence measurements at canopy and leaf scales under different nitrogen treatments [54]. Wang et al. introduced the state of the art of SIF measurement systems and the statistical methods for retrieval SIF on the canopy [55]. Frankerberg et al. published a review that introduced the origins of SIF, its relation to photosynthesis, and SIF retrieval at the canopy and global scale comprehensively [56]. Aasen et al. concluded the passive SIF measurement setups, protocols, and its application at the leaf to canopy level [57]. Cendrero-Mateo et al. wrote a review about the introduction and assessment of SIF retrieval methods for proximal sensing [57]. Gu et al. introduced SIF and the relationship between SIF and photosynthesis from the view of light reactions [58]. These reviews introduced the generation, retrieval, and applications of SIF from different views. Different from these reviews, this paper introduces SIF from the near-ground to global scale and summarizes the existing SIF retrieval methods for quick understanding. In this article, chlorophyll fluorescence estimation methods are collated and summarized. First, chlorophyll fluorescence measurement near the ground is introduced. Next, SIF retrieval algorithms from satellite data are introduced in detail. Finally, we summarize the existing problems and conclusions in future research. 

## 2. The Generation of Chlorophyll Fluorescence and Its Spectrum

Chlorophyll fluorescence is the production of chlorophyll excited by natural sunlight; chlorophyll is the essential pigment in the process of photosynthesis. Chlorophyll fluorescence, heat dissipation, and photosynthesis are important ways to consume the energy absorbed by the leaf (i.e., chlorophyll fluorescence has a direct relationship with photosynthesis). 

**Figure 1:**
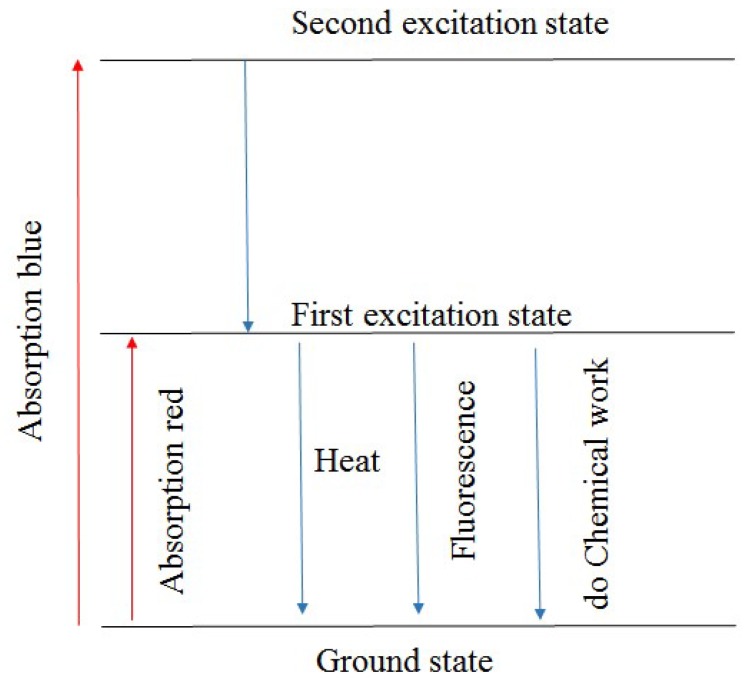
Generation of chlorophyll fluorescence, shows that chlorophyll molecules on the excited state release energy to return to the ground state through heat dissipation, photosynthesis, and fluorescence [59].

Vegetation mostly absorbs the visible light. When vegetation absorbs red light, chlorophyll molecules are excited to the first singlet state; when vegetation absorbs blue light, chlorophyll molecules are excited to the second singlet state (Figure 1). Chlorophyll molecules, which are in an unstable state, need to release the energy to return to a stable state. Heat dissipation is the key way of energy consumption. When chlorophyll molecules in the first singlet state, excluding the heat dissipate, photosynthesis and fluorescence are important approaches to dissipate the energy. From the first singlet state to the ground singlet state, chlorophyll molecules consume energy for chlorophyll fluorescence emission, which has a longer wavelength. 

**Figure 2:**
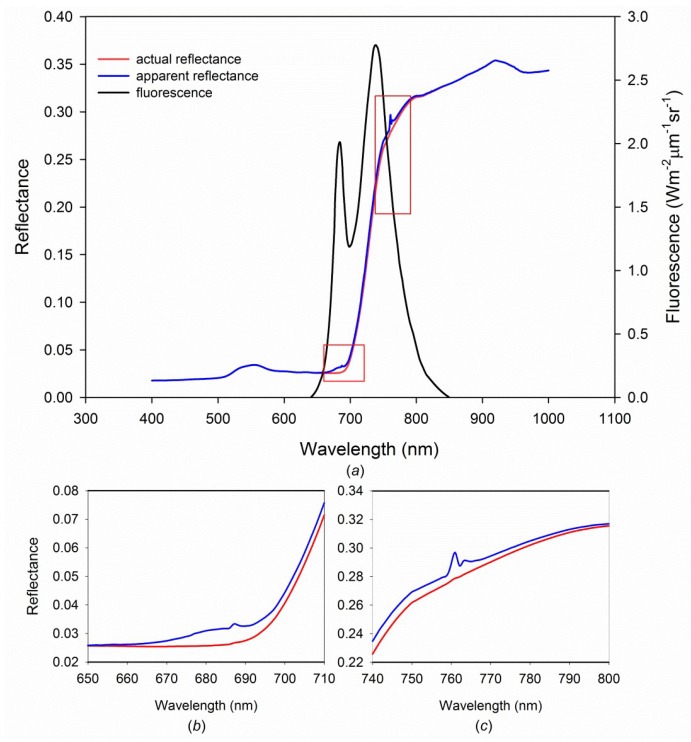
Chlorophyll fluorescence spectrum simulated by FluorMOD (the input parameters of FluorMOD are in default values). (**a**) The reflectance and fluorescence spectrum; (**b**) one convex at 690 nm in the actual reflectance; (**c**) one convex at 740 nm in the actual reflectance

The chlorophyll fluorescence spectrum ranges from the 640 nm to 850 nm and has two peaks (690 nm and 740 nm). By comparing the vegetation reflectance spectrum (the apparent reflectance) with the fluorescence-filtered vegetation reflectance spectrum (the actual reflectance) simulated by FluorMOD [60], the two convex can be found at 690 nm and 740 nm (Figure 2). 

## 3. Chlorophyll Fluorescence Detection near the Ground

### 3.1. Active Chlorophyll Fluorescence Measurements 

The active methods exploit the chlorophyll fluorescence kinetics to measure the relative parameters, including the modulated method (pulse-modulated chlorophyll fluorimeter [25,26,27,28,29,30]) and non-modulated method (non-modulated fluorimeter [31,32]). The modulated and non-modulated chlorophyll fluorimeters are designed based on the Kautsky effect [53,61,62,63]. 

A modulated fluorimeter uses the modulation measuring light in the whole process. When the measuring light from a modulated fluorimeter has the same frequency as the fluorescence, the fluorescence values can be recorded in every physiological state, including under the strong light. Therefore, a modulated fluorimeter can be used in field experiments. The world’s first pulse amplitude modulation (PAM) fluorimeter was designed and manufactured by Ulrich Schreiber (1983). Chlorophyll fluorescence induction dynamics parameters measured by PAM can reflect the variation of the vegetation [64] and are commonly used to detect the physiological state of the vegetation [25,26,28,29,30,54,65]. In addition, the chlorophyll fluorescence parameters are often combined with other related vegetation physiological parameters (stomatal conductance, leaf water potential, and so on) to analyze vegetation stress [66,67]. 

Non-modulated fluorimeters are designed to utilize a fast data acquisition system in order to record Kautsky induction or fast chlorophyll fluorescence induction [61], such as the Pochet plant efficiency analyzer (PEA), handy PEA, and multi-function PEA. 

Two different measurement methods can be used to obtain the chlorophyll fluorescence kinetic parameters. Non-modulated fluorimeters have a simple structure and are easy to operate. Continuous light is used in the whole measurement process. By contrast, modulated fluorimeters have a modulated light source to obtain chlorophyll fluorescence measurements in every physiological state. In fact, modulated fluorimeters are commonly used to detect vegetation stress [66,68].

### 3.2. Passive Chlorophyll Fluorescence Measurement

Passive chlorophyll fluorescence measurements use the filling of chlorophyll fluorescence in the Fraunhofer lines or oxygen absorption bands to retrieve the fluorescence information. The sun-induced fluorescence (SIF) retrieved on top of the canopy does not consider the atmospheric effects between the surface vegetation and the sensor, thus, the retrieval methods require solar irradiance and target radiance in two channels. These SIF retrieval methods near the ground are summarized in existing illustrations [57,69], and are divided into reflectance-based methods [28,70,71,72,73] and radiance-based methods, including the Fraunhofer line depth (FLD) [24,74], 3-band FLD [75], corrected FLD [76,77], improved FLD [78,79], extended FLD (eFLD) [80], and spectral fitting method (SFM) [69]. Reflectance-based methods mainly build the index using several spectral channels in the range of 650–800 nm to qualitatively reflect the fluorescence information. However, radiance-based methods are developed based on the FLD principle and can be used to obtain fluorescence radiation which has physical meaning. 

The FLEX mission is to accurately reconstruct the full fluorescence spectrum. Based on the SFM method, several full-specrum spectral fitting methods have been proprosed to reconstruct the SIF spectrum from top-of-canopy (TOC) measured data, such as fluorescence spectrum reconstruction (FSR) [81], full-spectrum spectral fitting method (F-SFM) [82], SpecFit method [83], and aFSR method [84].

#### 3.2.1. Spectral Fitting Method (SFM)

The canopy radiance can be expressed as the combination of the fluorescence contribution (SIF) and the reflectance radiance [85]:

(1) $$L(\lambda )=SIF(\lambda )+\rho (\lambda )\frac{E^{g}(\lambda )}{\pi }$$

In the selected bands of interest, such as the oxygen absorption bands or Fraunhofer lines, the least squares fitting technique is used to estimate the fluorescence and reflectance (assuming the reflectance and fluorescence are functions of wavelength), and the mathematical equation is expressed as Equation (2), and the variables are defined in Table 1:

(2) $$\min\sum {\left(L_{measure}(\lambda )-SIF(\lambda )-\rho (\lambda )\frac{E^{g}(\lambda )}{\pi }\right)}^{2}$$

Compared with other methods, the SFM method exploits more bands and a continuous spectrum to improve the signal-to-noise ratio (SNR) and is used to retrieve chlorophyll fluorescence from the FLEX-like simulate data [86] and FLEX/FLORIS simulated data [84]. The SFM method is developed to reconstruct the full SIF spectrum. Cogliati et al. proposed the SpecFit method to obtain the full SIF spectrum, and he selected a different combination of Gaussian, Lorentzian, and Voigt profiles to model the SIF emission peaks, and obtain the SIF spectrum by using the cubic spline fitting method which minimizes Equation (2) [83,87]. 

#### 3.2.2. Fluorescence Spectrum Reconstruction (FSR) Method and Advanced Fluorescence Spectrum Reconstruction (aFSR) Method

In Equation (1), Zhao et al. believed that reflectance and fluorescence can be expressed by Taylor polynomials at absorption lines [81]:

(3) $$\begin{array}{l} SIF(\lambda )\approx SIF(\lambda_{0})+\frac{dSIF(\lambda_{0})}{d\lambda }\cdot (\lambda -\lambda_{0})+\frac{1}{2}\cdot \frac{d^{2}SIF(\lambda_{0})}{d\lambda^{2}}\cdot {\left(\lambda -\lambda_{0}\right)}^{2}\\ \;=b_{0}+b_{1}\cdot (\lambda -\lambda_{0})+b_{2}\cdot {\left(\lambda -\lambda_{0}\right)}^{2}\\ \rho (\lambda )\approx \rho (\lambda_{0})+\frac{d\rho (\lambda_{0})}{d\lambda }\cdot (\lambda -\lambda_{0})+\frac{1}{2}\cdot \frac{d^{2}\rho (\lambda_{0})}{d\lambda^{2}}\cdot {\left(\lambda -\lambda_{0}\right)}^{2}\\ \;=b_{3}+b_{4}\cdot (\lambda -\lambda_{0})+b_{5}\cdot {\left(\lambda -\lambda_{0}\right)}^{2} \end{array}$$

Combining Equations (1) and (3), the canopy radiance can be expressed as follows [82]:

(4) $$L(\lambda )={\left(\lambda -\lambda_{0}\right)}^{2}\cdot \frac{E_{g}(\lambda )}{\pi }\cdot b_{5}+(\lambda -\lambda_{0})\cdot \frac{E_{g}(\lambda )}{\pi }\cdot b_{4}+\frac{E_{g}(\lambda )}{\pi }\cdot b_{3}+{\left(\lambda -\lambda_{0}\right)}^{2}\cdot b_{2}+(\lambda -\lambda_{0})\cdot b_{1}+b_{0}$$

in which, there are six unknown parameters. Through sampling at five absorption lines (Hα 656 nm, O_2_-B 687 nm, water vapor 719 nm, O_2_-A 761 nm, water vapor 823 nm), the unknown parameters are determined, then SIF radiance at absorption lines are obtained. In the second step, SIF data simulated by the SCOPE (Soil Canopy Observation, Photochemistry and Energy fluxes) model is used to generate the basis spectra of the full SIF spectrum by singular value decomposition (SVD), and the full SIF spectrum is written as follows [81]:

(5) $$SIF_{FSR}=c_{1}\cdot \upsilon_{1}+c_{2}\cdot \upsilon_{2}+c_{3}\cdot \upsilon_{3}$$

In Equation (5), *c*_1_, *c*_2_ and *c*_3_ are the coefficients of basis spectra, and are determined with the optimization process at five absorption bands. 

The FSR method only uses the information at five absorption bands of the SIF spectrum. Based on this idea, Zhao et al. proposed an aFSR (advanced FSR) method which uses the full information of the SIF spectrum. The upwelling radiance is expressed as follows [84]:

(6) $$L(\lambda )=\sum_{i=1}^{Nf}c_{fi}\phi_{fi}(\lambda )+\frac{E^{g}(\lambda )}{\pi }\sum_{i=1}^{Nr}c_{rj}\phi_{rj}(\lambda )$$

in which, the parameter descriptions are shown in Table 1. With respect to Equation (6), the linear least squares method is used to obtain the coefficients by computing the residual between the measured upwelling radiance and the modeled upwelling radiance in the range of 640–850 nm. In the last, the full fluorescence spectrum is constructed. 

The F-SFM method proposed by Liu et al. has a similar idea with the aFSR method [82]. In the F-SFM method, the reflectance is written as a first-order linear expression, and the basis spectra of the SIF spectrum is generated by principal components analysis (PCA).

#### 3.2.3. Radiative Transfer Model Inversion

In addition, Celesti et al. proposed a novel approach to explore the information of SIF and vegetation biochemical and biophysical parameters from the canopy-level high-resolution apparent reflectance data using the numerical inversion of the SCOPE model [88]. In the SCOPE model, the radiative transfer modules are used to simulate the reflectance and fluorescence [89]. Based on the radiative transfer modules of the SCOPE model, the apparent reflectance can be expressed as follows [88]:

(7) $$\rho^{*,\mathrm{RTM}}=\frac{\frac{r_{so}(\pi L_{sun}^{meas})+r_{do}(\pi L_{sky}^{meas})}{\pi }+F_{out}^{RTM}}{L_{sun}^{meas}+L_{sky}^{meas}}$$

in which, the first term of numerator is the modeled reflected radiance, the denominator is the incoming radiance. In fact, $L_{sun}^{meas}$ and $L_{sky}^{meas}$ are obtained by MODerate resolution atmospheric TRANsmission (MODTRAN). The cost function f is defined, and the least squares algorithm is used to obtain the full spectrum of canopy SIF [88].

(8) $$\begin{array}{l} f={\mathrm{ER}1}^{T}\mathrm{ER}1+\omega *{\mathrm{ER}2}^{T}\mathrm{ER}2\\ \mathrm{ER}1=\{\rho^{*,\mathrm{RTM}}(\lambda )-\rho^{*,\mathrm{meas}}(\lambda ),\;\lambda \in \lambda_{noabs}\\ \;\{(\rho^{*,\mathrm{RTM}}(\lambda )-\rho_{\mathrm{BL}}^{*,\mathrm{RTM}}(\lambda )\;)-(\rho^{*,\mathrm{meas}}(\lambda )-\rho_{\mathrm{BL}}^{*,\mathrm{meas}}(\lambda )\;),\;\lambda \in \lambda_{abs}\\ \mathrm{ER}2=\frac{p-p_{0}}{\sigma_{p0}} \end{array}$$

In Equation (8), the first term shows the two residuals, one is between the model reflectance and the measured apparent reflectance of the absorption bands, and the other one is residual of the height of the spikes due to the filling of the SIF between the modeled data and the measured data. The second term shows the priory knowledge and expected deviation. This method uses the radiative transfer modules of SCOPE to model the apparent reflectance, and then computes the residual between the modeled data and measured data to obtain the full spectrum of SIF. Verhoef et al. also used radiative transfer modeling to retrieve the SIF and other biophysical parameters [90]. Parameters used in Equations (7) and (8) are explained in Table 2.

## 4. SIF Retrieval Methods in Space Scale

The weak signal of chlorophyll fluorescence compared with the reflected signal makes it difficult to detect chlorophyll fluorescence from space, and it is about 2–5% of the reflected radiance in the near infrared spectral region. Numerous efforts have been made to decouple SIF from the reflected signal. The steady-state fluorescence at 685 nm was about 1.5–3.4 mW∙m^−2^∙sr^−1^∙nm^−1^, and that at 740 nm was 2.4–5.4 mW∙m^−2^∙sr^−1^∙nm^−1^ [91,92,93,94]. How to retrieve the SIF from the reflected signal is an important issue. In the past few years, numerous approaches have been proposed to retrieve SIF from the radiance received by a sensor. In short, these methods have two characteristics, one mainly using a physical method, and the other exploiting a statistical method.

The SIF retrieval algorithms based on the physical model are developed using the radiative transfer theory in the visible-near-infrared region. The assumptions are that both the surface reflectance and the fluorescence follow Lambert’s law and that the surface is uniform. Under these assumptions the radiation transmission equation can be simplified and fitted in the retrieval window to obtain the fluorescence radiance [33,36,38,44,45]. In addition, differential optical absorption spectroscopy (DOAS) is used to retrieve the fluorescence [46,47,48]. The fluorescence inversion algorithm based on a physical model has a clear physical meaning and a simple inversion process. However, the estimation of the atmospheric influence at the Fraunhofer line needs to be improved.

A statistical retrieval algorithm uses statistical methods, such as principal component analysis (PCA) [95] or singular value decomposition (SVD) [43,49,50], to estimate atmospheric effects and fit the fluorescence radiance in the spectral region of interest. These algorithms select the oxygen absorption bands or Fraunhofer lines for the medium spectral resolution data as the retrieval window. The wide window can improve the signal–to–noise ratio and reduce the sensitivity of the algorithm to the sensor noise. In the oxygen absorption band, the main atmospheric effects including atmospheric scattering and oxygen absorption, are estimated using statistical models to avoid computing the relative atmospheric parameters. Currently, most algorithms use the O_2_-A band to estimate the near infrared fluorescence [34,37,38,40,51], and some algorithms use the O_2_-B band to estimate the red fluorescence [96,97]. 

### 4.1. The Principle of Satellite SIF Retrieval Methods 

The satellite SIF retrieval methods are more complicated than the above-mentioned two platforms due to atmospheric effects. The SIF signal is very weak, and it can be easily affected by the atmosphere. It has been proved that SIF can be retrieved from the oxygen absorption bands or Fraunhofer lines, in which the proportion of SIF increases compared with other spectral regions. Therefore, these two spectral ranges are often used for the retrieval window.

The radiation received at the sensor consists of four components: (1) atmospheric path radiance, (2) the sun and sky irradiation reflected by the target, (3) the sun and sky irradiation reflected by the background, and (4) SIF radiance on the canopy. 

Assuming fluorescence and reflectance emission are isotropic, the radiance in the top of atmosphere (TOA) will be expressed as the addition of atmospheric contribution, surface-reflected radiance and the contribution of fluorescence signal. In Equation (9) [33,38,39,41,51,98], the meaning of the parameters are introduced in Table 3.

(9) $$L_{TOA}=\frac{E_{0}\cos\theta }{\pi }\rho_{so}+\frac{E_{0}\cos\theta }{\pi }\frac{\tau_{↑}\rho \tau_{↓}}{1-S\;\rho }+\frac{SIF\tau_{↑}}{1-S\;\rho }$$

The solar irradiation at the surface is affected by absorption and scattering effects related to atmospheric gases and aerosols. In our wavelengths of interest (600–800 nm), the main absorbers are oxygen (O_2_) and water vapor (H_2_O), and some narrow bands without the effect of oxygen and water vapor are used as retrieval window [47]. 

### 4.2. The Physical Methods

#### 4.2.1. FLD-Like Methods

The FLD algorithm is successfully used near the ground without considering the atmospheric effects. FLD-like methods are often combined with MODTRAN to extract the SIF from airborne or space-borne data [86,99,100,101,102]. 

Following Equation (9), the expression can be written in another form in the interested window:

(10) $$\begin{array}{l} L=L^{P}+\frac{E^{g}\cdot \rho /\pi +SIF}{1-S\cdot \rho }\cdot \tau_{↑}\\ L^{P}=\frac{E_{0}\cos\theta }{\pi }\rho_{so}\\ E^{g}=E_{0}\cos\theta \cdot \tau_{↓}\cdot S \end{array}$$

in which, $L^{P}$ is the atmospheric path radiance, $E^{g}$ is the irradiance including the direct and diffuse fluxes arriving at the surface. Other parameters are described in Table 3. The oxygen absorption band is chosen for the retrieved window. Two bands, one inside oxygen absorption band (i: 760 nm), and the other outside (o: 753 nm), are expressed as follows [86]:

(11) $$\begin{array}{l} L_{i}=L^{P}{}_{i}+\frac{E^{g}{}_{i}\cdot \rho_{i}/\pi +SIF_{i}}{1-S_{i}\cdot \rho_{i}}\cdot \tau_{↑}{}_{i}\\ L_{o}=L^{P}{}_{o}+\frac{E^{g}{}_{o}\cdot \rho_{o}/\pi +SIF_{o}}{1-S_{o}\cdot \rho_{o}}\cdot \tau_{↑}{}_{o} \end{array}$$

Combining the two previous equations, SIF can be calculated as follows [86]:

(12) $$SIF_{i}=B\left[\frac{X_{i}(E^{g}{}_{o}+\pi X_{o}S_{o})-AX_{o}(E^{g}{}_{i}+\pi X_{i}S_{i})}{B(E^{g}{}_{o}+\pi X_{o}S_{o})-A(E^{g}{}_{i}+\pi X_{i}S_{i})}\right]$$

(13) $$\begin{array}{l} X_{j}=\frac{L_{j}-L_{j}^{p}}{\tau_{↑j}},j=i,o\\ \rho_{i}=A\cdot \rho_{o}\\ SIF_{i}=B\cdot SIF_{o} \end{array}$$

A is the factor relating $\rho_{i}$ to $\;\rho_{o}$, *B* is the factor linking $SIF_{i}\;$ with $SIF_{o}$. Assuming the reflectance and fluorescence have a linear relationship in the oxygen absorption bands without considering the variation of spectral shape, A can be computed through the linear interpolation of surface reflectance through two channels (758 nm and 771 nm) outside the oxygen absorption band (760 nm) (Equation (7)) [86]; B is fixed at 0.8 [80,103] using field and simulated analysis.

(14) $$A=\frac{\rho_{758}\cdot \omega_{1}+\rho_{771}\cdot \omega_{2}}{\rho_{758}},\;\omega_{1}=\frac{771-760}{771-758},\;\omega_{2}=\frac{760-758}{771-758}$$

in which, ω_1_ shows the proportion of the right shoulder to the total width of the absorption band; ω_2_ shows the proportion of the left shoulder.

This method develops from the measurement near the ground and has a clear physical meaning; however, it needs to know the accurate atmospheric parameters. As we know, the atmospheric parameters are difficult to obtain, and are mainly obtained through simulation using the radiation transfer model. Therefore, these methods have been applied in the limited range and are not fit for measuring the SIF with conventional methods.

#### 4.2.2. Differential Optical Absorption Spectroscopy (DOAS)

The DOAS method is designed to measure the specific narrow-band absorption structures of trace gases for the UV and visible spectral range and determine the gas concentrations [104]. Considering chlorophyll fluorescence is a trace gas, the DOAS equation is rewritten in the following form [47], and the parameters are introduced in Table 4:

(15) $$-\ln\frac{L(\lambda ,\theta )}{E^{g}(\lambda ,\theta )}=\sum_{n=1}^{N}\sigma_{n}^{\prime }(\lambda )S_{n}+\sigma_{Ray}(\lambda )S_{Ray}+\sigma_{Mie}(\lambda )S_{Mie}+\sigma_{f}(\lambda )S_{f}+\sum_{m=1}^{M}a_{m}\lambda^{m}$$

In the DOAS method, $\sigma_{f}\left(\lambda \right)$ is considered as a pseudo-emission across the section, while $S_{f}$ acts as a fluorescence column relative to the emission cross section [47]. Through this hypothesis, the fluorescence can be obtained. Due to the lack of deep absorption features of oxygen and water vapor near the Fraunhofer lines, a 745–758 nm fitting window was chosen in Khosravi’s study [47]. Rayleigh and Mie scattering are removed using a low-degree polynomial that is also fitted. Equation (15) can be simplified as follows [47]:

(16) $$-\ln\frac{L(\lambda ,\theta )}{E^{g}(\lambda ,\theta )}=\sigma_{f}(\lambda )S_{f}+\sum_{m=1}^{M}a_{m}\lambda^{m}$$

In Equation (16), *L* and *E^g^* are known, $\sigma_{f}\left(\lambda \right)$ is the fluorescence spectrum, *m* is set to 3, and the coefficients $a_{m}$ and $S_{f}$ are fitted using the least squares algorithm. 

This method needs at least one fluorescence spectrum. The fluorescence input spectrum, which can be obtained through simulation, has an effect on the result. On the right of Equation (16), the first term is small compared with the last term. This case can result in a large error.

#### 4.2.3. The Fraunhofer Lines Depth Method

Considering litter atmospheric effects in the narrow Fraunhofer lines, the fluorescence is only one focus item. Under this condition, the unambiguous and accurate retrieval of fluorescence can be achieved. Frankenberg et al. computed the Fraunhofer line depth near the oxygen absorption bands using the following equation [36], and the meaning of parameters are introuduced in Table 5:

(17) $$\vec{f}(F_{s}^{rel},\alpha )=\log\left(\langle {\vec{I}}_{0}+F_{s}^{rel}\rangle \right)+\sum_{i=0}^{n}\alpha_{i}\cdot \lambda^{i}$$

It can be accepted that the effects of atmospheric scattering and surface albedo, which only affect the low-frequency part, can be expressed as a polynomial term in the Fraunhofer lines. In the narrow interesting bands, the fluorescence is thought of as a scalar and is wavelength-independent. Through the nonlinear weighted least squares algorithm [36], the parameters ($F_{s}^{rel},\alpha$) can be obtained. 

(18) $$\arg\min{\left\|s_{\in }^{-1/2}(\vec{y}-\vec{f}(F_{s}^{rel},\alpha ))\right\|}_{2}$$

In Equation (18), when $I_{0}\;$ is the transmission spectrum, $F_{s}^{rel}$ is unitless, and other parameters are explained in Table 5; then, other work should be done in order to obtain the fluorescence with a physical meaning ($F_{s}=F_{s}^{rel}/\left(1+F_{s}^{rel}\right)\cdot R_{cont}$) (Rcont is the continuum radiance).

#### 4.2.4. Simplified Radiative Transfer Method

Equation (9) gives the expression of the radiance at sensor. In some special window, such as the potassium (K) I absorption line, the CaII line near 866 nm, and some narrow spectrum including the Fraunhofer lines, the atmospheric scattering and absorption may be neglected (*ρ*_so_ = 0, *ρ* = 0, *τ*_ss_ + *τ*_sd_ = 1,*τ*_do_ + *τ*_oo_ = 1); the radiance received by the sensor can be simplified as follows [33]:

(19) $${\left(L_{TOA}\right)}^{*}={\left(\frac{\rho \cdot E_{0}\cdot \cos\theta }{\pi }+SIF\right)}^{*}=K\cdot E^{*}+SIF$$

in which, *E* is the high-resolution solar irradiance spectrum from Kurucz; the asterisk * shows that it is convoluted with respect to the instrumental line shape.

Similarly, in the range of interest, atmospheric scattering and absorption are considered constant values, and the received radiation is rewritten in the following form [33,44]:

(20) $${\left(L_{TOA}\right)}^{*}=K^{\prime }\cdot E^{*}+\varepsilon \cdot SIF$$

In Equations (19) and (20), *L_TOA_* and $E^{*}$ are known parameters, and other unknown parameters can be obtained through a standard, weighted least squares fitting method. These two equations show that neglecting the atmospheric effects only result in a slight scale factor ε. Some researchers proved that this scale factor was about 0.3, which can result in a 0.6% error [33]. In previous literature, K I lines from the GOSAT TANSO-FTS data, which have a super-fine spectral resolution, were mostly used as the retrieved window [33]. To improve the SNR, the retrieved window will be widened (i.e., 769.9–770.25 nm including the K I line, 758.45–758.85 nm near the oxygen absorption band, and 863.5–868.8 nm including the CaII line) [44]. With respect to GOSAT data, the other simplified radiative transfer method GARLiC was proposed by Köhler [38]. The retrieval window was 755–759 nm, and the upward transmittance on a clear day was 1, and *ρ* was much smaller than 1; therefore, Equation (9) can be simplified as follows [38]:

(21) $$L_{TOA}=\frac{E_{0}\cdot \cos\theta }{\pi }\cdot (\rho_{so}+\rho \cdot \tau_{↓})+SIF$$

In Equation (21), under the assumption that the atmospheric scattering and surface reflectance are functions of the wavelength, this expression can be further simplified as follows [38]:

(22) $$L_{TOA}=E^{*}\cdot (\alpha_{0}+\alpha_{1}\cdot \lambda )+SIF$$

in which, $\alpha_{0}$ and $\alpha_{1}$ are the parameters describing the effects of atmospheric scattering and surface reflectance, respectively. Lastly, the least squares fitting method is applied to obtain $\alpha_{0},\;\;\alpha_{1}$, and *SIF*.

The physical methods are commonly used in the fluorescence retrieved. All these methods have a clear physical meaning; but they were developed from different strategies. In these methods, the atmospheric effects can be neglected. However, in future research, the atmospheric effects should be paid more attention. 

### 4.3. The Statistical Methods 

#### 4.3.1. Singular Value Decomposition (SVD)

Based on the concept of SVD, Guanter believed that radiance without SIF could be written as the linear summation of several singular vectors [34]. Therefore, the radiance received by a sensor is the composition of SIF-free radiance and SIF radiance at the top of the atmosphere. The equation can be written as [34] and parameters defination are in Table 6:

(23) $$F(\omega ,F_{s})=\sum_{i=1}^{n_{v}}\omega_{i}\nu_{i}+SIF^{TOA}I$$

In this method, it is important to obtain the singular vector. Following certain rules, the SIF-free objects were selected and trained to generate the singular vector. In Equation (23), no items showed the effects of atmospheric scattering and absorption. Therefore, the strong absorption band should be removed from the selected window to maintain the retrieved accuracy. In Equation (23),$\;n_{v}$, weights $\omega_{i}$, and $F_{S}^{TOA}$ were unknown parameters and were fitted through linear least squares. 

When the interest window enlarges, it may include some wavelengths that are affected by atmospheric scattering, vegetation structure, and other factors. Guanter modified the previous SVD methods to cope with this lower frequency information, and applied this method to the GOSAT data [51]. The SVD approach had been successfully used in TanSat chlorophyll fluorescence retrival [43].

#### 4.3.2. Principal Component Analysis (PCA)

Based on Equation (9), it is assumed that the effects of atmospheric scattering are neglected in the limited window ($\rho_{so}≃0$ and S·ρ≪1). The reflectance is expressed as follows [37]:

(24) $$\rho_{tot}=\tau_{↑}\cdot \rho \cdot \tau_{↓}+\frac{\pi \cdot SIF\cdot \tau_{↑}}{E_{0}\cdot \cos\theta }$$

In Equation (24), $\tau_{↑}$, $\tau_{↓}$, and SIF are the unknown parameters, and the target parameter is SIF. To remove the unknown parameters, sun-to-satellite (two-way) atmospheric transmittance τ(λ) is used and defined as the production of $\tau_{↑}$ and $\tau_{↓}$ ($\tau \left(\lambda \right)=\tau_{↑}*\;\tau_{↓}$). Through the mathematic relationship between them, the upward transmittance $\tau_{↑}\;$ is written as follows (*θ*: sun zenith angle, *θ*_0_: view zenith angle) [37]:

(25) $$\tau_{↑}(\lambda )=\exp\left[\ln\tau (\lambda )\frac{\sec\theta_{0}}{\sec\theta +\sec\theta_{0}}\right]$$

With the help of several simplifications, the reflectance equation is as follows [37]:

(26) $$\rho_{tot}=\rho \cdot \tau +\frac{\pi \cdot SIF}{E_{0}\cos\theta }\cdot \exp\left[\ln\tau (\lambda )\frac{\sec\theta_{0}}{\sec\theta +\sec\theta_{0}}\right]$$

Within the limited spectral fitting window, SIF is considered a Gaussian function of wavelength centered at 736.8 nm with a standard deviation of 21.2 nm, *ρ* is expressed as a low-degree polynomial wavelength, and τ(λ)  is expressed using principal components (PCs), which are trained from the satellite data. Previous work suggests that different descriptions of the fluorescence spectrum have very little effect on the retrieval results [51,98]. 

In this method, the atmospheric effects were considered, and solved through the PCA method. GOME-2 and SCIAMACHY data were used to retrieve the fluorescence using the PCA methods [37,39]. This method can be used in the total fluorescence spectrum including both red and far-red features. 

## 5. Current Problems and Discussion

Significant progress has been made in chlorophyll fluorescence remote sensing from the leaf scale to the satellite scale using active and passive remote sensing technology. Today, chlorophyll fluorescence is widely used in research on the correlation with the physiological state of vegetation. Chlorophyll fluorescence detection has achieved good results at the leaf scale and at the canopy scale and has also been used effectively to monitor vegetation water stress [29,30,42,72,73,93,105,106,107,108,109,110,111], ozone stress [66,112,113], nitrogen stress [3,4,54,114,115,116,117,118], pest stress [119,120], GPP [14,15,16,17,18,19,20,21,22,23,121,122,123,124], heat stress [125], and crop productivity [126]. Despite many experiments designed to clarify the relationship between chlorophyll fluorescence and vegetation stress, the internal mechanisms of this relationship remain to be explored. For sun-induced chlorophyll fluorescence derived from the satellite data, the atmospheric effects are significant and make retrieval more complicated. The problems associated with the retrieval methods under consideration will be discussed briefly below.

### 5.1. The Treatment of Atmospheric Effects

The radiance received by a sensor is affected by atmospheric conditions, azimuth information of the sun/sensor, and so on. In our wavelengths of interest (600–800 nm), the main absorbers are oxygen (O_2_) and water vapor (H_2_O) [47,127]. With respect to the atmospheric effects, scattering is the primary factor that should be considered in the SIF retrieval approaches. In the Fraunhofer lines, SIF will adequately fill the Fraunhofer well and scattering also contributes to the Fraunhofer well. Many studies about the effects of scattering on the SIF retrieval method have been conducted [61,128,129,130,131,132], and conclude that Raman scattering should be considered in the space-sacle SIF retrieval method [47].

In SIF retrieval methods, two retrieval windows are used, which are oxygen absorption bands and Fraunhofer lines. In the oxygen absorption bands, Raman scattering, surface pressure, albedo, and so on may produce errors in the algorithms [37]. SIF retrieval methods usually exploit statistical techniques to calculate the atmospheric effects in the SIF retrieval process, such as PCA [37] and SVD [34]. Fluorescence-free regions, such as deserts, Greenland, Antarctica, and so on, are used for training to estimate the influence of atmospheric effects. The types and number of the trained data must be as representative as possible. By training many fluorescence-free datasets, the computed atmospheric condition can be made more reliable. The proper selection of fluorescence-free regions is critical [34]. 

In the Fraunhofer lines, the atmospheric effects are assumed to be very small, and can be neglected [33]. Based on this assumption, the SIF retrieval methods in the Fraunhofer lines are developed. Yet until now, how the neglection of atmospheric effects in Fraunhofer lines give the effects on SIF is not clear. Contrasting with the received radiance, the fluorescence radiance is relatively weak (approximately 1–3% are strong). Inappropriate treatment of the atmospheric effects can affect the fluorescence retrieval results.

### 5.2. The Zero-Level Offset Correction 

Fluorescence of the non-vegetation regions is zero. In fact, because of rotational-Raman scattering and the disadvantages of the various retrieval methods, non-vegetation regions, such as the Sahara Desert, exhibit non-zero fluorescence values [33]. These fluorescence values are thought of as fluorescence bias; all retrieval methods should remove the fluorescence bias to obtain the reliable values. Frankerberg et al. found that non-linearity problems exist in the TANSO-FTS band 1 and proposed an empirical method to correct the resulting fluorescence by expressing fluorescence offset as a function of the average at-sensor radiance over Antarctica [36]. Based on this idea [36], Guanter et al. [34], Joiner et al. [44], and Köhler et al. [38,39] increased the vegetation-free objects as reference spectra and employed a strict criterion for selecting reference spectra, such as the range of the fluorescence values and the average radiance, as well as the sun zenith radiance. The threshold values of these parameters have no common standard and were determined by the researchers according to the feature of the sensor. These fluorescence offset correction strategies select the vegetation-free regions as the target and build the relationship between the fluorescence offset and the average radiance, and the vegetation areas with the same average radiance as vegetation-free regions are thought to have the same fluorescence offset. Joiner et al. [97] suggested that the previous fluorescence offset correction methods do not consider dark current, stray light, and nonlinear responses, and developed an empirical correction scheme to mitigate zero-level offsets. The reasons for the formation of zero-level offsets are complicated and remain to be studied in the future. New zero-level offset correction methods have not yet been developed.

### 5.3. Lack of Surface Data for Validating Sun-Induced Chlorophyll Fluorescence Derived from the Satellite Data

The SIF derived from satellite data validation is a problem in current research. The most commonly used methods are cross-validated through other SIF products, such as OCO-2 SIF, GOSAT SIF, GOME-2 SIF and SCIAMACHY SIF, or NDVI data [9,13,33,34,35,37,38,39,40,42,97,102,124]. The lack of surface measurement data results in less support for the fluorescence product, which may limit its future. The SIF is considered a useful probe for detecting the condition of vegetation. It has a close correlation with photosynthesis, thus it is used to derive GPP. Uncertainties in the fluorescence observation due to variation in the sun-satellite view observation geometry can affect GPP estimation [133,134]. The number of fluorescence measurements made near the ground is small, and the measurement range is limited. Thus, it is difficult to validate SIF satellite data using surface data. To validate SIF satellite data, it is essential to expand the ground fluorescence observation network. 

## 6. Conclusions and Perspectives

Based on the presented fluorescence retrieval methods and current problems, some recommendations for the estimation of chlorophyll fluorescence are proposed below.

### 6.1. Research over the Atmospheric Effects on the Fluorescence Retrieval 

Since atmospheric effects are not considered, the SIF can be successfully used to monitor the vegetation at the leaf or canopy scale. SIF retrieved from the airborne/space-borne images still faces challenges. Thus, the retrieval of SIF in these situations needs to correct atmospheric effects [35,86]. SIF detection from satellite data is a hot research topic and is regarded as a critical mission of the FLEX project. In recent years, SIF detection research has also achieved much progress, and SIF has been successful retrieved from satellite data, such as SCIMACHY, GOSAT, GOME-2, OCO-2, TROPOMI, and TanSat. Using the features of the interest window, these methods neglect the atmospheric effects or compute these effects via statistical methods. Although atmospheric effects, such as aerosol scattering and surface pressure, in fluorescence retrieval methods are frequently considered, other factors still require further analysis [86], including rotation Raman scattering (RRS) and stray light. These atmospheric effects result in less significant filling than the fluorescence in the oxygen absorption bands or Fraunhofer lines, but they still induce fluorescence retrieval errors. 

Given the weak fluorescence signal, atmospheric effects should be considered fully and deeply. In recent years, more people focused on research regarding the atmospheric effects on SIF estimation. Daumard et al. believed that the transmittance of an air column, the path radiance, and the adjacency effect are three main factors that affect the oxygen absorption band depth; they used MODTRAN 4 to compute the atmospheric and environmental parameters and then corrected the measured airborne radiance to obtain ground-level oxygen absorption bands relating to the SIF [135]. Sabater et al. analyzed how atmospheric effects impact SIF retrieval on proximal sensing (at tower scale) by using simulated data with MODTRAN and provided a rigorous oxygen compensation method by introducing the oxygen transmittance function into the FLD or SFM to improve SIF estimation [136]. Liu et al. also estimated the upward and downward atmospheric transmittances using MODTRAN to obtain the downwelling irradiance and upwelling radiance at the canopy, and then retrieved the SIF by the 3-band FLD method [137]. Celesti et al. [88] and Verhoef et al. [90] used the radiative transfer model inversion method to estimate the SIF. In the report for mission selection (an earth explorer to observe vegetation fluorescence), it stated that it was essential to process the atmospheric correction to mitigate error propagation in retrieved SIF, and it is believed that the atmospheric correction process mainly considered the presence of aerosols and the total atmospheric columnar water vapor (CWV) in oxygen absorption bands, which was carried out by the retrieval of aerosol and water vapor to derive the apparent reflectance, and then the full fluorescence spectrum through the SFM method was retrieved [138,139]. In future research, more attention will be paid to the SIF retrieval based on rigorous atmospheric correction. 

### 6.2. Constructing the Fluorescence Validation Network

Due to the limited development of fluorescence remote sensing, the availability of fluorescence ground measurement data is very limited, making it difficult to use fluorescence data to validate the airborne or space-borne fluorescence results. The fluorescence retrieval methods based near the ground without considering atmospheric effects have been successfully applied in numerous studies. Thus, the fluorescence values near the ground can be thought of as ‘true’ values and can be utilized to validate other fluorescence retrieval results. Using standard spectral measurement and fluorescence retrieval technology, the ground fluorescence measurement network was constructed in order to obtain the ‘true’ fluorescence value. With the emergence of more fluorescence satellite data and products, it is urgent that we validate the fluorescence satellite data in order to improve fluorescence satellite retrieval methods and accuracy.

Chlorophyll fluorescence varies with vegetation biochemical parameters and canopy structure; thus, it has a different response to vegetation species under different environmental conditions. The fluorescence validation network construction covering a large number of measured samples will ensure the global validity of SIF data. In the FLEX fluorescence project, the “bottom–up” scheme was proposed to validate the FLEX fluorescence products, which start from tower-based canopy fluorescence measurements to the landscape level including the different vegetation types and the non-vegetated surfaces. Several factors, such as measured sites, vegetation types, structures, phenology, the range of photosynthetically active radiation (PAR), and so on, should be considered. Based on the existing data sites, such as FLUXNET, the deployment of fluorescence measurements may be carried out [138,139].

## Figures and Tables

**Table 1:** Parameters used in the Equations (1)–(6).

Parameter	Meaning
*L*(λ)	The canopy radiance
*L*_measure_(λ)	Measured canopy radiance
*SIF*(λ)	Fluorescence radiance
ρ(λ)	Canopy reflectance
$E^{g}(\lambda )$	The integral of incoming radiance over hemisphere in the bottom of atmosphere
$c_{fi}$	Coefficients of basis spectra of fluorescence
$c_{rj}$	Coefficients of basis spectra of reflectance
$\phi_{fi}(\lambda )$	Basis spectra of fluorescence
$\phi_{rj}(\lambda )$	Basis spectra of reflectance
Nf	The number of basis spectra of fluorescence
Nr	The number of basis spectra of reflectance
*b*_0_, *b*_1_, *b*_2_, *b*_3_, *b*_4_, *b*_5_	Coefficients of the expressions of solar-induced fluorescence (SIF) and reflectance
*SIF*_FSR_	The full SIF spectrum
*c*_1_, *c*_2_, *c*_3_	Coefficients of basis spectra
*v*_1_, *v*_2_, *v*_3_	Basis spectra of full SIF spectrum

**Table 2:** Parameters used in Equations (7) and (8).

Parameter	Meaning
$\rho^{*,RTM}$	The modeled apparent reflectance
$r_{so}$	Bi-directional reflectance of target
$L_{sun}^{meas}$	Solar irradiance
$L_{sky}^{meas}$	Sky irradiance
$r_{do}$	Hemispherical-directional reflectance factor of target
$F_{out}^{RTM}$	Modeled fluorescence in the observation direction
$\rho^{*,\mathrm{meas}}$	The measured apparent reflectance
$\rho_{\mathrm{BL}}^{*,\mathrm{RTM}}$	The modeled baseline reflectance inside the absorption band
$\rho_{\mathrm{BL}}^{*,\mathrm{meas}}$	The measured baseline reflectance inside the absorption band
$\lambda_{noabs}$	The band between 400–900 nm
$\lambda_{abs}$	Spectral ranges within the 640–850 nm
p	The posterior value of the model parameters
$p_{0}$	The priori values of the model parameters
$\sigma_{p0}$	The expected standard deviation
*f*	The cost function

**Table 3:** The definations of parameters used in Equation (9).

Parameter	Meaning
*L*_TOA_	Radiance at the top of atmosphere
$\rho_{so}$	Hemispherical reflectance
$E_{0}$	extraterrestrial solar irradiance on a plane perpendicular to the sun’s rays
θ	Solar zenith angle
ρ	Surface reflectance
SIF	Fluorescence radiance at the top-of-canopy (TOC)
S	Spherical reflectance of the atmosphere back to the surface
$\tau_{↑}$	Upward transmittance
$\tau_{↓}$	Downward transmittance

**Table 4:** Parameters used in Equation (15).

Parameter	Meaning
*S_n_*	The density of the absorber
${\sigma^{\prime }}_{n}\left(\lambda \right)$	Rapid part of the absorption cross section of the absorber
N	Number of absorbers
$\sigma_{Ray}\left(\lambda \right)$	Reference spectra of Rayleigh scattering
$\sigma_{Mie}\left(\lambda \right)$	Reference spectra of Mie scattering
$\sigma_{f}\left(\lambda \right)$	Reference spectra of fluorescence
$\overset{M}{\underset{m=1}{\sum }}a_{m}\lambda^{m}$	Low-order polynomial, in which $a_{m}$ is the coefficient of the polynomial, and $\lambda^{m}$ is the wavelength.
*S_f_*	Fluorescence fit factor

**Table 5:** Parameters used in Equations (17) and (18).

Parameter	Meaning
$\vec{I_{0}}$	High-resolution solar transmission spectrum
$F_{s}^{rel}$	Relative fluorescence signal
< >	Convolution symbol (with the instrumental line shape)
$\overset{n}{\underset{i=0}{\sum }}a_{i}\lambda^{i}$	Polynomial item (the continuum radiance), in which $a_{i}$ is the coefficient of the polynomial, $\lambda^{i}$ is the wavelength.
$\vec{y}$	Logarithm of the measurement vector
$S_{\epsilon }$	Diagonal measurement error covariance matrix

**Table 6:** Parameter in Equation (23).

Parameter	Meaning
$v_{i}$	The singular vector
$\omega_{i}$	The weight of the singular vector $v_{i}$
SIF^TOA^	Fluorescence intensity at the top of the atmosphere
I	An identity vector of size *n*
$n_{v}$	The number of singular vectors
$F(\omega ,F_{s})$	The radiance at the sensor
